# RNA isolation from Peyer’s patch lymphocytes and mononuclear phagocytes to determine gene expression profiles using NanoString technology

**DOI:** 10.14440/jbm.2018.246

**Published:** 2018-07-02

**Authors:** Navjot Singh, Heather C. Gallagher, Renjie Song, Jaskiran K. Dhinsa, Gary R. Ostroff, Magdia De Jesus

**Affiliations:** 1 Division of Infectious Diseases, Wadsworth Center, New York State Department of Health, Albany NY, USA; 2 Division of Molecular Genetics Wadsworth Center, New York State Department of Health, Albany NY, USA; 3 Biochemistry and Immunology Core, Wadsworth Center, New York State Department of Health, Albany NY, USA; 4 Department of Biomedical Sciences, University at Albany, School of Public Health Albany, Albany NY, USA; 5 Program in Molecular Medicine, University of Massachusetts Medical School, Worcester, MA, USA

**Keywords:** B-lymphocytes, mononuclear phagocytes, Peyer’s patches, NanoString technology, T-lymphocytes

## Abstract

Sampling and immune surveillance within gut-associated lymphoid tissues such as the intestinal Peyer’s patch (PP) occurs by an elegantly orchestrated effort that involves the epithelial barrier, B and T lymphocytes, and an extensive network of mononuclear phagocytes. Although we now understand more about the dynamics of antigen and microbial sampling within PPs, the gene expression changes that occur in individual cell subsets during sampling are not well characterized. This protocol describes the isolation of high-quality RNA from sorted PP, B and T-lymphocytes, and CD11c^+^ phagocytes for use with nCounter-NanoString technology. This method allows investigators to study gene expression changes within PPs in response to antigens, microbes, and oral vaccine delivery vehicles of interest that are sampled.

## BACKGROUND

In addition to its absorptive functions, the small intestine is faced with a two-fold challenge; it must tolerate incoming antigens and microbes while serving as a site of immune surveillance [1]. This carefully orchestrated immune surveillance takes place within aggregate lymphoid structures called Peyer’s patches (PPs) [1]. Selective transcytosis of luminal antigens, microbes, and oral vaccine delivery vehicles into PPs occurs through specialized enterocytes called microfold cells (M-cells) that are interspersed within the epithelial barrier called the follicle-associated epithelium (FAE) [2-6]. Below the FAE, in the sub-epithelial dome (SED), there is an extensive and dynamic network of dendritic cell (DCs) and macrophage subsets. A decade ago, Iwasaki and Kelsall identified three distinct (CD11c^+^) DC subsets within PPs, including the lymphoid DCs (CD11b^-^CD8α^+^) that reside in the interfollicular regions (IFR), the myeloid DCs (CD11b^+^ CD8α) localized in the SED, and the “so-called” double negative (DN) DCs that lack both CD11b^-^ and CD8α^-^ surface expression and localize to the SED and IFR [7]. More recently, lysozyme-expressing DCs (Lyso DCs) have also been identified in the SED, as well as DCs that express the C-type lectin Langerin [7-10]. These DC subsets in the SED capture and present antigens to resident T- and B-lymphocytes, and are also known to influence specific T-helper (Th) responses [7,11,12]. Although we understand more about the mechanisms of how antigen and microbial sampling occur, the gene expression changes that take place within specific PP immune cells during sampling are not well characterized [10,13]. Recently, Bonnardel and colleagues developed a method to isolate and perform transcriptional analysis on PP mononuclear phagocyte subsets such as the CD11b^+^ conventional DCs, the lysozyme-expressing monocyte-derived DC termed LysoDC, and the CD11c(hi) lysozyme-expressing macrophages [14-16]. Bonnardel’s method demonstrates that there is much value in studying different PP cell subsets using transcriptomics [14,16].

To gain insight into the gene expression changes of specific PP cells at baseline and in response to vaccine delivery vehicles, we have developed a protocol that isolates high-quality RNA from specific PP cells such as B- and T-lymphocytes and CD11c^+^ phagocytes. Total RNA isolated from sorted cells is then subjected to NanoString nCounter^®^ technology that uses a novel digital color-coded molecular barcode technology to measure gene expression based on the counts of the target RNA [17]. In these experiments, we specifically used the nCounter^®^ PanCancer immune profiling array panel to gain insight into the gene expression levels of over 770 genes within PPs by interrogating B- and T-lymphocytes and CD11c^+^ phagocytes. To compare gene expression changes at baseline levels, we used Saccharomyces cerevisiae-derived β-glucan particles (GPs). Earlier studies using GPs demonstrated that these particles are taken up by both PP M-cells and CD11c^+^ phagocytes and are a good positive control as they are known to stimulate an immune response [10,18,19]. It is important to note that PP cells are extremely delicate and the RNA is prone to rapid degradation; therefore, stabilization of RNA early in this procedure is critically important. This method allows for the investigator to reproducibly isolate stable RNA from sorted B- and T lymphocytes and CD11c^+^ phagocytes to determine gene expression changes using any antigen, microbe or delivery vehicle of interest in a murine model.

## MATERIALS

### Animals

Swiss Webster mice (8–12 weeks old) were obtained from Taconic Farms (Hudson, NY). Animals were housed under conventional, specific pathogen-free conditions and were treated in compliance with the Wadsworth Center’s Institutional Animal Care and Use Committee (IACUC) guidelines. We recommend usage of age-matched mice for consistency to reduce outlier variability.

### Ethics statement

Experiments described in this study that involve mice were reviewed and approved by the Wadsworth Center’s Institutional Animal Care and Use Committee (IACUC) under protocol # 15-450. The Wadsworth Center complies with the Public Health Service Policy on Humane Care and Use of Laboratory Animals and was issued assurance number A3183-01. Moreover, the Wadsworth Center is fully accredited by the Association for Assessment and Accreditation of Laboratory Animal Care (AAALAC). Obtaining this voluntary accreditation status reflects that Wadsworth Center’s Animal Care and Use Program meets all of the standards required by law, and goes beyond the standards as it strives to achieve excellence in animal care and use.

### Reagents

- RNase Zap Decontamination Solution (Thermo Fisher Scientific, cat. # AM 9780)
- 70% Ethanol (Pharmco, cat. # 04343-19)
- β-glucan particles (GPs) [20]
- β-glucan particles with residual mannan content (GMPs) [20]
- β-Mercaptoethanol (Sigma, cat. # M6250)
- 0.4% Trypan Blue (Amresco, cat. # K940)
- RNeasy Mini Kit (Qiagen, cat. # 74106)
- DNase RNase-Free Set (1500 Kunitz units) (Qiagen, cat. # 79254)
- SUPERase·In RNase Inhibitor (20 U/µl) (Thermo Fisher Scientific, cat. # AM 2694)
- Agilent RNA 600 Pico Kit (Agilent Technologies, cat. # 5067-1513)
- Agilent RNA 600 Nano Kit (Agilent Technologies, cat. # 5067-1511)
- nCounter Low RNA input Amplification kit (Nanostring, cat. # LOW-RNA-48)
- Target enrichment Primer pool (NanoString, cat. # PP-MIP1-12)
- nCounter^®^ PanCancer Immune Profiling Kit (NanoString, cat. # GXA-PATH1-12)
- Rat anti-mouse CD45R/B220 PerCp Clone RA3-6B2 (BD Pharmigen, cat. # 553093)
- Rat anti-mouse CD3 FITC-Clone 17A2 (BD Pharmigen, cat. # 555274)
- Rat anti-mouse Fc-Block CD16/CD32-Clone 2.4G2 (BD Pharmigen, cat. # 553142)
- Armenian Hamster anti-mouse CD11c-Clone N418 (eBioscience, cat. # 17-0114-82)
- Luna Universal qPCR Master Mix (New England Biolabs, cat. # M3003X)
- ProtoScript II First Strand cDNA Synthesis Kit (New England BioLabs, cat. # E6560S)
- DNA Oglios (IDT, # See **Fig. S1** for sequences)
- Gold Taq Flexi DNA polymerase (Promega, cat. # M8295)
- Deoxynucleotide (dNTP) Solution Set (New England BioLabs, cat. # N0446S)
- 100 bp DNA Ladder (Thermo Fisher, cat. # 10488058)
- Equipment
- Kleen Guard Gloves (Kimberly Clark, cat. # 57370)
- Straight Surgical Scissor (FST, cat. # 14060-09)
- Curved Surgical Scissor (FST, cat. # 14061-09)
- 70 µm nylon cell strainer (CELLTREAT, cat. # 229483)
- 12 × 75 mm Polystyrene Tubes with Cell Strainer Cap (BD, cat. # 352235)
- Cell Strainer Pestle (CELLTREAT, cat. # 229480)
- Countess Cell Counter (Thermo Fisher Scientific, cat. # C10281)
- Countess Cell Counting Chamber Slides (Thermo Fisher Scientific, cat. # C10312)
- Squared Petri Dishes (Fisher Scientific, cat. # FB0875711A)
- 22 G × 1.5 in blunt-end feeding needle (Popper Scientific, New Hyde Park, NY)
- Fetal Bovine Serum (Thermo Fisher, cat. # A3160401)
- Spin-X Centrifuge Tube 0.22 µm Filter (Costar, cat. # 8161)
- 2% E-gel (Thermo Fisher Scientific, cat. # G501802)
- iBase (Thermo Fisher Scientific, cat. # G6400)
- Freeze Zone 4.5 Benchtop Lyophilizer (Labconco, cat. #7750020)
- 96 Well Thermal iCycler (Biorad, cat. # 170-8740)
- Chip Priming Station (Agilent, cat. # 5065-4401)
- Vortex Mixer (IKA, cat. # 0025001607)
- 16 pin bayonet electrode cartridges (Agilent, cat. #5065-4413)
- PCR Machine DNA Engine Dyad (MJ Research, cat. # PTC-220)
- 2100 Expert Software (Agilent, Version B.02.08.SI648 (SR2))
- nCounter Prep Station Version 4.1.0.1
- nCounter digital analyzer Version 4.0.0.3
- nSolver analysis software 3.0
- FACSAria IIu Flow Cytometry Sorter (BD, cat. # 643895)
- BD FACSDiva Flow Cytometry Sort/Analysis Softer Ware Version 6.1.3 (BD, cat. # 643629)
- BD Falcon 5 ml Polystryrene Round-Bottom Tube with Cell-Strainer Cap (BD, cat. # 32235)

### Recipes

***Hanks’ balanced salt solution without sodium bicarbonate and phenol red pH 7.2 (1 L, Sigma cat. # H1387)***

8 g NaCl, 0.4 g KCl, 0.06 g KH2PO_4_, 0.048 g NaHPO_4_,1 g D-Glucose, 0.098 g MgSO_4_ (anhydrous), 0.14 g CaCl_2_ (anhydrous), Adjust the pH to 7.2 with HCl

***1× phosphate buffered saline pH 7.4 (1 L)***

8 g of NaCl, 0.2 g of KCl, 1.44 g of Na_2_HPO_4_, 0.24 g of KH_2_PO_4_, Adjust the pH to 7.4 with HCl

***Flow buffer***

1× phosphate buffered saline pH 7.4, 2% fetal bovine serum

## PROCEDURE

### Oral gavage

***1.*** Gavage mice with phosphate buffered saline (PBS) or with antigen of interest. In these experiments, we used 1 × 10^8^ highly purified, Saccharomyces cerevisiae derived β-glucan particles (GPs) as a positive control. As a second positive control, 1 × 10^8^ purified β-glucan particles with exposed mannans (GMPs) were used in 200 µl of 1× PBS. Delivery of particles was achieved using a 22 G × 1.5 in blunt-end feeding needle. Both GPs and GMPs are known to stimulate the immune response [19,21].
  **NOTE:** Delivery volumes should not exceed 400 µl per mouse.

### Isolation of total Peyer’s patch cells from mice

***2.*** Before euthanizing mice, prepare one tube containing 2 ml of Hanks’ balanced salt solution (HBSS) with 50 µl of SUPERase·In RNase inhibitor (20 U/µl), label as tube number 1, and place it on ice. Label one additional tube as number 2, add 2 ml of HBSS with 30 µl of SUPERase·In RNase Inhibitor (20 U/µl) and place on ice.
  **NOTE:** This step is critical, as addition of the SUPERase·In RNase inhibitor immediately stabilizes RNA when PPs are harvested. Without the SUPERase·In RNase inhibitor, RNA recovery will be poor. Keeping everything on ice is extremely important.
  **CAUTION:** DO NOT USE RNAlater RNA Stabilizer Reagent (Qiagen cat. #76104) specifically for PPs, as this reagent will significantly reduce the viability of PP cells to 40%. The RNA quality was also found to be very poor. We found that RNAlater made PPs float in solution during the harvesting step, suggesting that these tissues were damaged. PPs naturally sink to the bottom of the tube in solution.
  ***2.1.*** Euthanize mice by CO_2_ asphyxiation, according to institutional guidelines.
  ***2.2.*** Cleanse the mouse abdomen with 70% ethanol prior to necropsy. Perform a standard necropsy that involves a 1 cm incision using straight surgical grade scissors along the midline beginning about 1.5 cm from the base of the rib cage. Expose the peritoneal cavity and identify the cecum. Snip the terminal small intestine at the ileal-cecal junction, gently remove the small intestine in its entirety, and snip the stomach-ileal junction to release intact intestine.
  ***2.3.*** Identify individual PPs located on the anti-mesenteric side of the intestine. Mice typically contain 5 to 10 PPs spaced more or less evenly from the duodenum (proximal) to ileum (distal).
  ***2.4.*** Using curved surgical scissors, gently excise individual PPs and place them immediately in cold HBSS that contains 50 µl of SUPERase·In RNase inhibitor (tube 1). The scissors should be placed curve-side up just above the PP and then gently applied to the tissue. Add RNAase Zap to surgical instruments and then rinse with ethanol between every mouse.
  **NOTE:** Two to three mice yield plenty of cells to be able to isolate RNA from all three PP cell types. To avoid cell clumping, isolate PPs from less than 5 mice per group.
  ***2.5.*** Decant HBSS in tube 1 and transfer PPs into tube 2 that contains 2 ml of HBSS with 30 µl of SUPERase·In RNase inhibitor.

### Staining cells for cell sorting

 
  **NOTE:** Before moving on to this step, wipe petri dish, pestle and cell strainer with RNase Zap decontamination solution.
***3.*** To generate a cell suspension, decant tube 2 into a 70 μm nylon mesh cell strainer that is resting on a petri dish. This setup should be on ice. Grind PPs with pestle or with the back part of a syringe plunger. The 2 ml should yield approximately 1200 µl after grinding the PPs. Transfer the cell suspension from the petri dish into a microfuge tube [21]. If you use RNAlater RNA Stabilizer Reagent instead of SUPERase·In RNase inhibitor, PPs will feel like rubber at this step and will not yield high quality RNA.
  ***3.1.*** Save 10–15 µl of cells to count them in step 3.5.
  **CRITICAL STEP:** Prepare flow buffer that contains SUPERase· In RNase inhibitor, as this buffer will be used for all subsequent steps to stain and wash cells for cell sorting. It is recommended to prepare 20 ml of flow buffer at a 1:250 dilution.
  ***3.2.*** Using a 96-round bottom plate, add 50 µl of cells per well for single color controls. For B-cells, T-cells and DCs that will be sorted, fill ten wells per cell type, with 100 µl of cells per well. Spreading cells into multiple wells prevents cell clumping in subsequent steps.
  ***3.3.*** Spin the plate for 2 min at 2100 rpm (887× g), to pellet the cells and decant supernatant by inverting the plate.
  ***3.4.*** Prepare a (1:100) dilution of Rat anti-mouse Fc-block in flow buffer and resuspend cells in 100 µl of this buffer and incubate on ice for 15 min. Place the ice bucket on a rocker during incubation.
  ***3.5.*** During the incubation step with the Fc block, count viable cells in the suspension by preparing a 1:20 dilution of cells with trypan blue. Add 10 μl of the cell suspension to the countess chamber slides and count cells. 20 PPs should yield approximately 1.5 × 10^6^ cells per ml with a viability range of 90%–98%.
  ***3.6.*** After the 15 min incubation, spin the plate for 2 min at 2100 rpm (887× g), and decant supernatant by inverting the plate.
  ***3.7.*** Resuspend the cells in 100 µl of fluorophore-conjugated antibodies added directly to cell suspensions (1:200 for B and T-cells and 1:40 for CD11c^+^ phagocytes) and incubate on ice for 30 min with continuous rocking.
  ***3.8.*** Spin the plate for 2 min at 2100 rpm (887× g), and decant supernatant by inverting the plate.
  ***3.9.*** Wash cells with 200 μl of flow buffer.
  ***3.10.*** Spin the plate for 2 min at 2100 rpm (887× g), and remove the supernatant by inverting the plate.
  ***3.11.*** Resuspend the cells from the plate into 3 ml of flow buffer; some of cells will clump. Pass the 3 ml cell suspension through a filtered cap flow tube that contains 1ml of flow buffer resulting in a total of 4 ml of cell suspension for cell sorting.

### Cell sorting of B- and T-lymphocytes and CD11c^+^ phagocytes and RNA isolation

***4.*** Cells were analyzed and sorted on a FACS Aria IIu cell sorter. Prior to the collection of sorted cells, a cell purity test sort should be performed to check that the cell types of interest are free of debris and contamination by other cell types in the sample.
  **NOTE:** Magnetic bead isolation yields mixed populations of cells, making it difficult to interpret NanoString data. A combination of magnetic bead isolation with cell sorting also decreases RNA yield significantly.
  **NOTE:** RNA samples derived from PPs are sensitive to degradation, even with the addition of RNase inhibitors. The RNeasy Mini Kit calls for cell wall lysis by using a syringe, which leads to RNA degradation. We therefore developed an alternative method for cell lysis using the Spin-X filter column (step 4.1).
  ***4.1.*** Sort CD45R/B220^+^(CD3^-^/CD11c^-^) B-cells, CD3^+^(CD45R/B220^-^/CD11c^-^) T-cells and CD11c^+^(CD45R/B220^-^/CD3^-^) CD11c^+^ phagocytes onto a filtered Spin-X column that is moistened with 100 µl of flow buffer that containing SUPERase·In RNase inhibitor. For PP B and T-cells we sorted 300000 cells. Since CD11c^+^ phagocytes are the least abundant of the three cell types in PPs, we sorted a maximum of 50000–80000 cells.
  ***4.2.*** During sorting, centrifuge the Spin-X column every 100000 cells at 2000 rpm for 1 min at room temperature (20°C to 30°C) as this is the maximum volume that the tubes hold during cell sorting. Discard flow buffer through and continue sorting.
  ***4.3.*** When sorting is finished, centrifuge the Spin-X column at 2000 rpm for 2 min. Place the filter in a new 1.5 ml collection tube. Discard flow buffer through an old collection tube.
  ***4.4.*** Add 350 µl of Qiagen’s RLT buffer that contains β-Mercaptoethanol (BME) (10 µl of BME in 1 ml of RLT) to the filter, pipette 20–25 times to mix. Place tube on dry ice for 5 min.
  ***4.5.*** Incubate on rotary spinner for 10 min and vortex at the end of the incubation. The RNA extraction is performed at room temperature (20°C to 30°C).
  ***4.6.*** Centrifuge the Spin-X column for 2 min at 13400 rpm. Keep flow-through. To obtain a maximum yield of RNA, the flow through is passed a second time unto the filter.
  ***4.7.*** Transfer the flow-through back onto the filter, and centrifuge for 2 min at 13400 rpm. Keep flow-through.
  ***4.8.*** Add 100 µl of RNase free H_2_O to the filter and pipette 20–25 times, and centrifuge for 1 min at 13400 rpm. Keep flow through and discard the filter.
  ***4.9.*** Add 250 µl 100% ethanol to the tube.
  ***4.10.*** Transfer the liquid to the RNeasy spin column and pipette 20–25 times, incubate for 5 min on bench top. Centrifuge for 1 min at 13400 rpm. Discard flow-through.

### Column DNase digestion

 
  **NOTE:** The DNase RNase-Free Set was used according to manufacturer’s instructions.
  ***4.11.*** Add 350 µl buffer RW1 to the RNeasy spin column. Close the lid gently, and centrifuge for 15 s at 13400 rpm to wash the spin column membrane. Discard the flow-through.
  ***4.12.*** Add 10 µl DNase I stock solution to 70 µl buffer RDD. Vortex to mix and add 80 µl directly to each RNeasy spin column membrane, then incubate on the bench top for 15 min.
  **NOTE:** Be sure to add the DNase I incubation directly to the RNeasy spin column membrane. DNase digestion will be incomplete if part of the mix sticks to the walls of the spin column.
  ***4.13.*** Add 350 µl buffer RW1 directly onto the RNeasy spin column that contains the 80 µl of DNase I and spin for 15 s at ≥ 10000 rpm. Discard the flow-through.
  ***4.14.*** Add 500 µl buffer RPE to the RNeasy spin column. Close the lid gently, and centrifuge for 15 s 13400 rpm to wash the spin column membrane. Discard the flow-through.
  ***4.15.*** Add 500 µl buffer RPE to the RNeasy spin column. Close the lid gently, and centrifuge for 2 min at 13400 rpm to wash the spin column membrane. Discard the flow-through.
  ***4.16.*** Add another 500 µl buffer RPE to the RNeasy spin column. Close the lid gently, and centrifuge for 2 min at 13400 rpm to wash the spin column membrane. Discard the flow-through.
  ***4.17.*** Place the RNeasy spin column in a new 2 ml collection tube and discard the old collection tube containing the flow-through. Spin at 13400 rpm for 1 min to eliminate any possible carryover of buffer RPE.
  ***4.18.*** Place the RNeasy spin column in a new 1.5 ml collection tube. Add 30 µl of RNase-free water directly to the spin column membrane, incubate for 2 min, then spin at 13400 rpm for 2 min to elute the RNA. Keep flow-through.
  ***4.19.*** Add 10 µl of RNase-free water directly to the spin column membrane, incubate for 2 min, then spin at 13400 rpm for 2 min to elute the RNA. Keep flow-through. Vortex the 40 µl of collected RNA.
  ***4.20.*** Sample can be stored short-term at **−**20°C; for longer storage, store at **−**80°C.

### Lyophilize RNA to concentrate

***5.*** RNA stored at **−**80°C must be placed on dry ice and the lid of the tube covered with parafilm. Make small holes on the top of the tube using a flame-heated needle and place inside a lyophilization flask. Lyophilize for 3 h. To ensure that the flask remains cold and the RNA remains frozen, place the flask on dry ice while it is attached to the lyophilizer. Resuspend lyophilized RNA in 6 µl of RNase-free water.

### Measurement of RNA using bioanalyzer

***6.*** Before use, thaw sample and ladder on ice until fully resuspended. Take 1 µl of RNA sample and ladder and heat-denature at 70°C for 2 min.
  ***6.1.*** Agilent RNA 6000 Pico kit protocol was followed as per manufacturer’s instructions.
  **NOTE:** The RNA yield for B-cells was in the range of 3–20 ng. For T-cells, the yield was in the range of 1.5–14 ng, and for CD11c^+^ phagocytes, the yield was 0.06–0.6 ng, in a total volume of 5 µl each. Prior to hybridization with the reporter and capture probe set, we used the nCounter Low RNA Input Amplification kit to generate cDNA followed by the multiplexed target enrichment primer pool to amplify target genes in the codeset. Since the yield of RNA is low, it is important to check the desired amplification of targets in all cell types by reverse transcription polymerase chain reaction (PCR) and by reverse transcription quantitative PCR before proceeding with nCounter Low RNA Input amplification.

### RT-PCR and RT-qPCR assays for detection of gene expression

***7.*** Synthesize the cDNA using ProtoScript II first strand cDNA Synthesis Kit from NEB using 1 ng of RNA for each cell type with random hexamer primers.
  ***7.1.*** Use gene-specific primers to perform either reverse transcription-PCR (RT-PCR) or quantitative RT-PCR (RT-qPCR) on the cDNA. RT-PCR was performed on cDNA to ensure the amplification efficiency and specificity of the primers. Using both methods, we chose three housekeeping genes from the immune profiling codeset Hprt, Tubb5 and Sf3a3 (**Fig. S1**).
  ***7.2.*** RT-PCR conditions: Initial denaturation 94°C for 3 min, 94°C for 20 s, 55°C for 20 s, 72°C for 20 s (30×) and 72°C for 5 min, followed by hold at 4°C. RT-qPCR conditions: Initial denaturation 95°C for 60 s, 95°C for 15 s and 60°C for 30 s (40×), followed by hold at 4°C.
  ***7.3.*** To estimate the minimum number of cycles needed for cDNA amplification, we used the RT-qPCR assay, using Luna Universal qPCR Master Mix and the primers for the housekeeping genes mentioned in 7.1.
  ***7.4.*** The estimated number of cycles from the RT-qPCR was used in the nCounter Low RNA Input Amplification assay.

### nCounter low RNA input amplification assay

***8.*** Add RNA (2 ng or less) with the provided reagents in the kit; 0.5 µl of 10× RT enzyme mix, 0.5 µl of 10× Primer Mix and bring up to 5 µl with RNase-free water.
  ***8.1.*** Synthesize cDNA in thermocycler using the following conditions: anneal primer at 25°C for 10 min, first strand cDNA synthesis at 42°C for 60 min, enzyme inactivation for 85°C for 5 min and hold at 4°C.
  ***8.2.*** Add the following to the cDNA: 1.5 µl 5× dT Amp Master Mix, 1 µl gene specific primers (500 nM per primer) corresponding to the codeset for multiplexed target enrichment. Gently, flick to mix and centrifuge.
  **NOTE:** Estimate the number of cycles to generate dsDNA for each cell type based on manufacturer’s instructions and the data collected during the RT-PCR/RT-qPCR in step 7.2.
  ***8.3.*** Set up the amplification reaction in thermocycler. Initial denaturation 95°C for 10 min, 95°C for 15 s and 60°C for 4 min, followed by hold at 4°C. Number of cycles needs to be estimated on cell type, as stated in the note.
  ***8.4.*** Proceed with NanoString hybridization or store the dsDNA at **−**80°C.

### Reading RNA counts with NanoString

***9.*** Thaw the dsDNA if frozen or proceed with hybridization.
  ***9.1.*** Heat denatures the dsDNA at 95°C for 2 min. Immediately cool on ice.
  ***9.2.*** Pre-heat PCR thermocycler to 65°C with the lid set to 70°C.
  ***9.3.*** Thaw reporter code set and capture code set on ice. Mix by flicking or inverting tubes.
  **NOTE:** DO NOT VORTEX. Mix by flicking or inverting tubes, followed by a quick spin in picofuge. Make sure to change pipette tip every time.
  ***9.4.*** Prepare master mix for the 12 samples. The master mix includes 70 µl of sample hybridization buffer to the provided reporter code set.
  **NOTE:** DO NOT VORTEX. Mix by flicking or inverting tubes, followed by a quick spin in picofuge. Make sure to change pipette tip every time.
  ***9.5.*** Label strip tubes and cut the strip in the middle to yield 6 tubes each. Aliquot 8 µl of master mix into strip tubes.
  ***9.6.*** Adjust dsDNA input to 5 µl in dH_2_O.
  **NOTE:** It is important to empirically adjust the input amount so that the concentration of dsDNA is not too high or too low.
  ***9.7.*** Add dsDNA to the 8 µl of master mix into labeled strip tubes.
  **NOTE:** DO NOT VORTEX. Mix by flicking or inverting tubes, followed by a quick spin in *picofuge*. Make sure to change pipette tip every time.
  ***9.8.*** Add 2 µl of capture probe set to every tube.
  **NOTE:** DO NOT VORTEX, Mix by flicking or inverting tubes, followed by a quick spin in *picofuge*. Make sure to change pipette tip every time.
  ***9.9.*** Close the tubes and immediately transfer to the 65°C thermocycler set up in step 9.2.
  ***9.10.*** Incubate in 65°C for 12–30 h.

### PREP station

 
  ***9.11.*** Warm sealed cartridge from **−**20°C to room temperature and sealed reagent plates from 4°C to room temperature (20°C–30°C).
  ***9.12.*** Centrifuge reagent plates 2000 g for 2 min.
  ***9.13.*** Follow prep station automated instructions until it requests sample loading. Immediately remove sample from 65°C, spin in Picofuge, carefully remove caps, and load in prep station. Immediately initiate protocol by pressing start.
  ***9.14.*** Remove cartridge and seal with adhesive tape to prevent evaporation. Tape is provided in kit.
  ***9.15.*** Take cartridge and place in digital analyzer for RNA copy counts.

### Digital analyzer

 
  ***9.16.*** Upload the Reporter Library file in the provided flash drive.
  ***9.17.*** Create a CDF file with sample name and description and upload into the digital analyzer.
  ***9.18.*** Insert cartridge with the seal (step 9.14) into the digital analyzer.
  ***9.19.*** Initiate counts by pressing start.
  ***9.20.*** When the digital analyzer is complete download your data for analysis.
  ***9.21.*** Follow instructions on nSolver or self-analyze.

## STATISTICAL ANALYSIS

NanoString analysis was performed in duplicate per experimental condition with the nCounter Analysis System using the **PanCancer Immune profiling kit**. The results were analyzed using the raw count with DEseq2 [22,23]. A transcript was considered differentially expressed when up or downregulated at least 2-fold and the *P*-adjusted value ≤ 0.05.

## ANTICIPATED RESULTS

**Figure 1.**
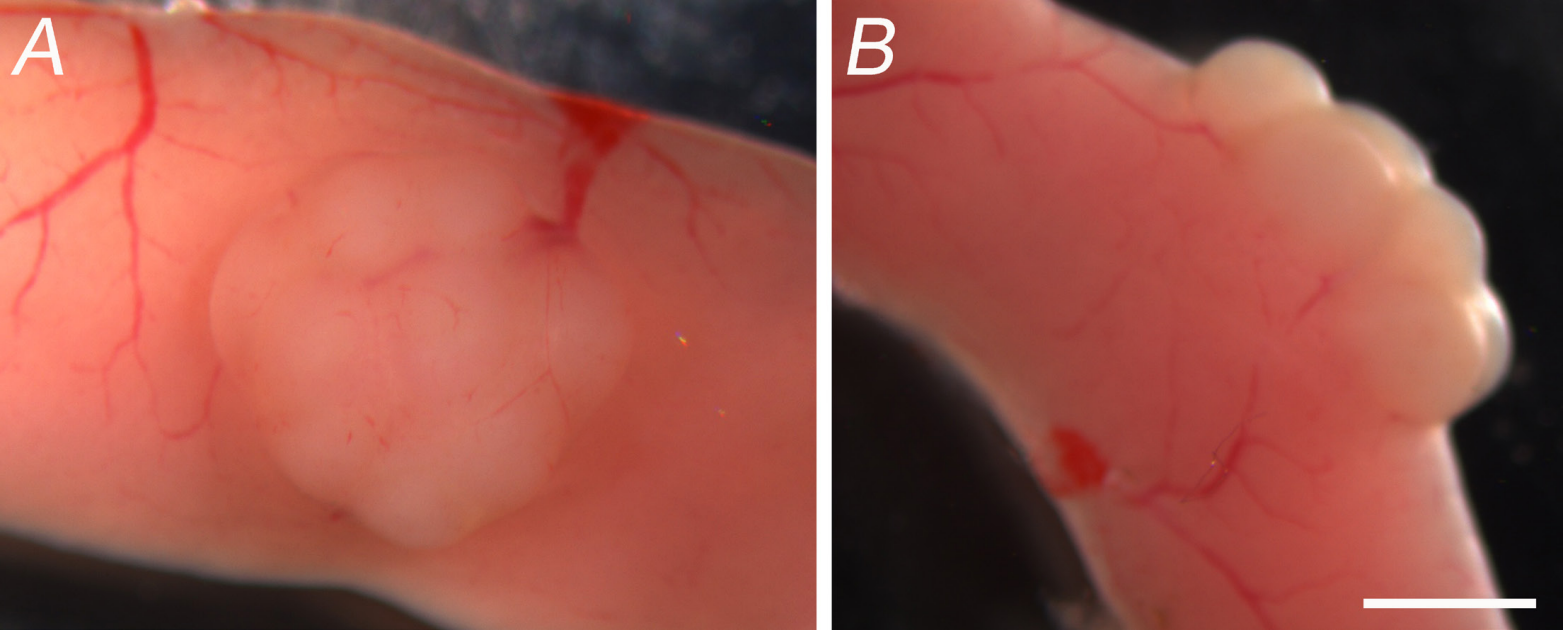
Peyer’s patches are aggregate lymphoid structures. **A.** Top view image of PP show that these are aggregate lymphoid structures that are on the anti-mesenteric side of the small intestine. **B.** Profile image of PP reveal multiple follicles, all of which participate in sampling and immune surveillance. PPs are surrounded by an extensive network of blood and lymphatic vessels that allow the dynamic movement of immune cells in and out of the gut. Scale bar is 1 mm.

PPs are aggregate lymphoid structures that can be easily seen with the naked eye and are located on the anti-mesenteric side of the small intestine (**Fig. 1A**). Each PP contain multiple follicles that are individually equipped with an epithelial barrier, an extensive network of mononuclear phagocytes, and T- and B-lymphocytes that play an important role in sampling luminal antigens, microbes, and oral vaccine delivery vehicles (**Fig. 1B**) [10]. To assess the transcriptomics of individual cell types in response to β-glucan delivery vehicles, PPs were harvested and RNA was isolated. It is important to note that isolation of PPs cells can be challenging because PP cells are extremely delicate and difficult to maintain as viability decreases by roughly 15% every 30 min [21]. Additionally, in the absence of RNA inhibitors, the RNA quality from PPs is extremely poor with less than 10% recovery. To overcome these challenges, we treated PP single cell suspensions with two different commercially available RNase inhibitors, RNAlater and SUPERase·In RNase inhibitor. We found that treatment with RNAlater caused PPs to float in solution, yielding a hard and tacky texture that was not ideal for making single cell suspensions. Cell viability using the RNAlater reagent was determined to be 46% (**Fig. S2**). We noted that PPs treated with SUPERase·In RNase inhibitor remained in the bottom of the tube, as expected, and single cell suspensions yielded 98% viability (**Fig. S2**). Most importantly, we found that viability of PP single cell suspensions remained above 90% after 2–4 h on ice with SUPERase·In RNase inhibitor, a significant improvement from our previous protocol, which was done without inhibitors [24].

**Figure 2.**
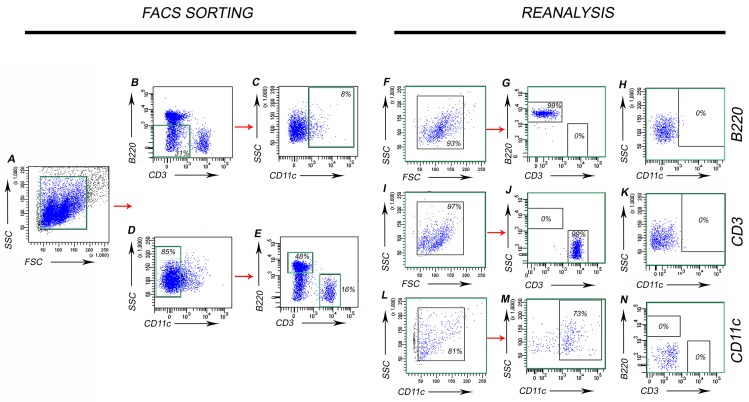
Cell sorting and reanalysis of isolated PP B-cells, T-cells and CD11c ^+^ phagocytes. Single cell suspension of PPs were stained for the cell surface markers: B220 for B-cells, CD3 for T-cells and CD11c for CD11c^+^ phagocytes. FACS sorting panels: **A.** Forward (FSC) and side scatter (SSC) plot represents the entire single cell suspension. Cells were gated on (B) B220^-^/CD3^-^ cells, which represented 31% of the population. **C.** CD11c^+^ phagocytes which represented 8% of the population. **D.** CD11c^-^ cells (representing 85% of the population). **E.** B220^+^ CD3^-^ cells (representing 48% of the population) and B220^-^ CD3^+^ cells (representing 16% of the population). Reanalysis panels: **F-H.** The purity for each cell type. For B220^+^ B-cells, a 99% purity was obtained. **I-K.** For CD3^+^ T-cells, a 99% purity was obtained. Neither B or T-cells were contaminated with CD11c^+^ phagocytes. **L-N.** For CD11c^+^ phagocytes, 73% purity was obtained; these also were not contaminated by B- or T-cells.

After determining which RNase inhibitor was optimal in prolonging cell viability and providing RNA stability, we used cell sorting to separate PP B220^+^ B-cells, CD3^+^ T-cells and CD11c^+^ phagocytes (**Fig. 2A**). Using this method, we sorted a single PP suspension of cells into three separate tubes that contained CD45R/B220^+^(CD3^-^/CD11c^-^) B-cells, CD3^+^(CD45R/B220^-^ /CD11c) T-cells and CD11c^+^(CD45R/B220^-^/CD3^-^) phagocytes. During sorting, we carefully selected the gates denoted in green (**Fig. 2A-2E**) such that there was no cross-contamination of each of the cell types of interest. Reanalysis of the sorted cells revealed that we accomplished 99% purity for B220^+^ B-cells (**Fig. 2G**), 99% purity for CD3^+^ T-cells (**Fig. 2J**), and 73% purity for CD11c^+^ phagocytes (**Fig. 2M**). Although we could not achieve a higher percentage of purity for CD11c^+^ phagocytes due to their complicated shape, we determined that there was no contamination by B220^+^ or CD3^+^ cells (**Fig. 2N**). We also found that using a combination of both magnetic bead cell isolation and cell sorting did not improve cell purity but, rather, it decreased RNA yield to 10%–15%, due to the increased experimental time and cell losses in each step (data not shown). Using both bioanalyzer nano and pico chips, we determined that RNA isolated using RNA-later was highly degraded (**Fig. S3A**, lanes 2–4). SUPERase·In RNase inhibitor alone yielded multiple bands. However, many of these bands were not clearly defined due to DNA contamination (**Fig. S3A**, lanes 6–8). RNA purified by SUPERase·In RNase inhibitor was of higher quality, although the preparation was contaminated with DNA. We therefore subjected the RNA preparation to DNase treatment, which resulted in DNA-free RNA. The bioanalyzer picochip results yielded reproducible bands that represent 28S (4.7 kb) and 18S (1.9 kb) rRNA (**Fig. S3B**, lanes 2–4). We also compared two RNA extraction methods by sorting cells directly unto Trizol reagent or Spin-X columns that contained a 0.22 µm membrane. Spin-X columns were subjected to dry ice and RNeasy reagents to extract RNA from cells. We determined that the Spin-X column RNA extraction method consistently yielded DNA-free RNA of high quality in the bioanalyzer data (**Fig. S3B** and data not shown). We also determined the limits of detection of the RNA isolated per cell type and per single mouse. We found that PP RNA isolation for B- and T -lymphocytes can be achieved at an individual mouse level as both are abundant within PPs. For CD11c^+^ phagocytes, RNA amounts fall below the limits of detection; therefore, more than one mouse must be used for this cell type (data not shown).

**Figure 3.**
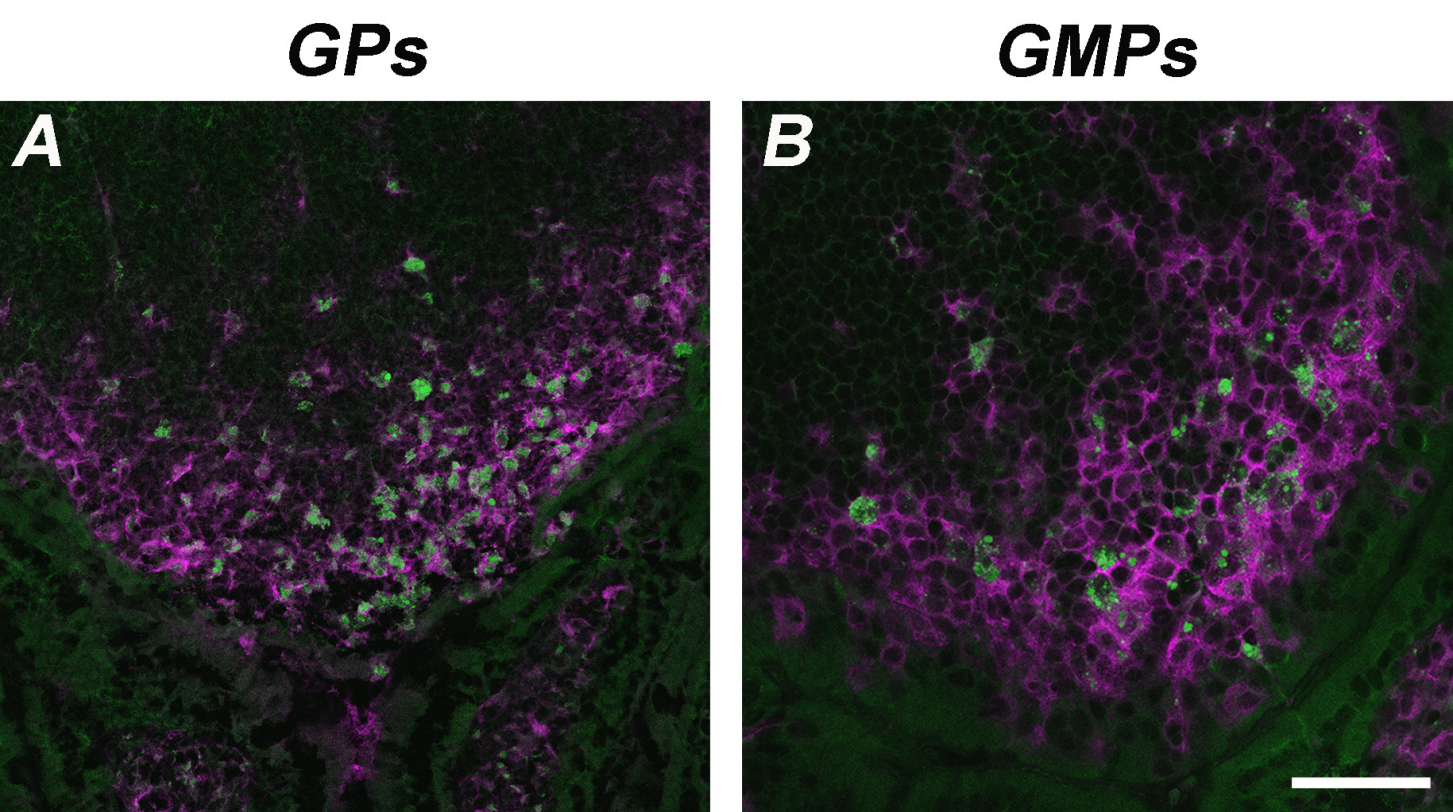
Comparison of GPs and GMPs 24 h post gavage. PPs from mice gavaged with FITC-labeledGPs (A) and GMPs (green) (B) were harvested after 24 h. Both GPs and GMPs are localized within CD11c^+^ phagocytes (magenta) in the SED of the patch. Scale Bar is 100 µm.

To assess the gene expression profiles of each cell type, we used the nCounter^®^ mouse Pan Cancer Immune profiling kit that profiles over 770 murine immune system-related genes. To obtain both reproducible and biologically relevant results from PP RNA, it was critical to start with high quality RNA. To compare the differential expression of genes per cell type in comparison to the untreated control, we gavaged mice with β-glucan particles (GPs) derived from *Saccharomyces cerevisiae* cell walls. GPs are highly purified, spherical hollow shells that mainly consist of β-1, 3-D-glucans. Because GPs are recognized by Dectin-1 and complement 3 receptors, they are considered useful antigen presenting cell-targeted vaccine adjuvant/delivery vehicles. The inner hollow cavity of GPs can be loaded to deliver antigens to macrophages and dendritic cells [20]. In addition to GPs, we also used GMPs, which are considered a lower purity β-glucan particle that contain residual mannan content in the particle cell wall. GMPs have also been shown to have immunostimulatory properties [19]. Confocal microscopy of PPs from mice gavage with GPs and GMPs show that both types of particles are sampled by PPs CD11c^+^ phagocytes (**Fig. 3A** and **3B**). Since GPs and GMPs can serve as both antigen-presenting cell-targeted delivery systems and as adjuvants, we were particularly interested in differential gene expression of CD11c^+^ phagocytes [20].

**Figure 4.**
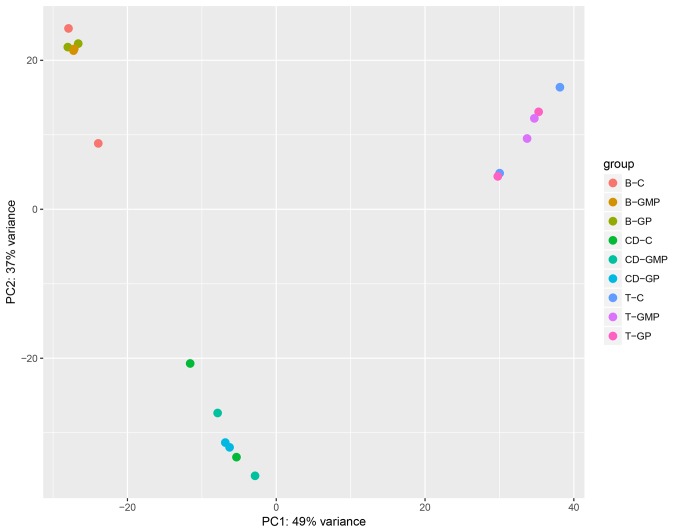
Principal component analysis of PP cell types. Principal component analysis demonstrates gene expression profiles are clustered based on cell types B-cells, T-cells and CD11c^+^ phagocytes, regardless of treatment with GP, GMPs or control.

To determine highly significant differential gene expression, we analyzed the RNA raw counts with DEseq2. Differential expression of transcripts was stringently defined, requiring transcripts to be up or downregulated at least 2-fold and the *P*-adjusted value to be ≤ 0.05. Mice were gavaged with GPs and GMPs, and PPs were harvested after 24 h. When comparing the differential expression within each cell type at baseline and with stimulation by GPs and GMPs, the results reveal that stimulation with GPs did not yield any significant differential gene expression in B-cells and T-cells. Only one gene, interleukin 1 receptor type 1, (IL-1r1), showed differential expression in CD11c^+^ phagocytes. In contrast, GMPs stimulated gene expression in CD11c^+^ phagocytes but not in B- and T-cells. The four genes that were most significantly upregulated in CD11c^+^ phagocytes by GMPs were interleukin-22 (IL-22), (21-fold), deleted in malignant brain tumors 1 (Dmbt1) 11-fold, IL-1r1 (6-fold), and Fibronectin-1 (Fn1) (6-fold). IL-22, is a key cytokine that has been demonstrated to induce antimicrobial activity, stimulating tissue-damage protection and tissue repair and remodeling [25]. The Dmbt1 gene encodes a glycoprotein with multiple scavenger receptor domains that may be involved in the activation of the complement mannose lectin pathway, and influences IL-22 [26]. IL-1r1 is the receptor for IL-1, a proinflammatory cytokine that influences IL-22 [27]. Fibronectin-1 (Fn1) is involved in cell adhesion and motility [28] (**Table 1**). In addition, principal component analysis of the data show that gene expression profiles are clustered based on cell types regardless of treatment, indicating that there are larger changes in gene expression between cell types (**Fig. 4**).

**Figure 5.**
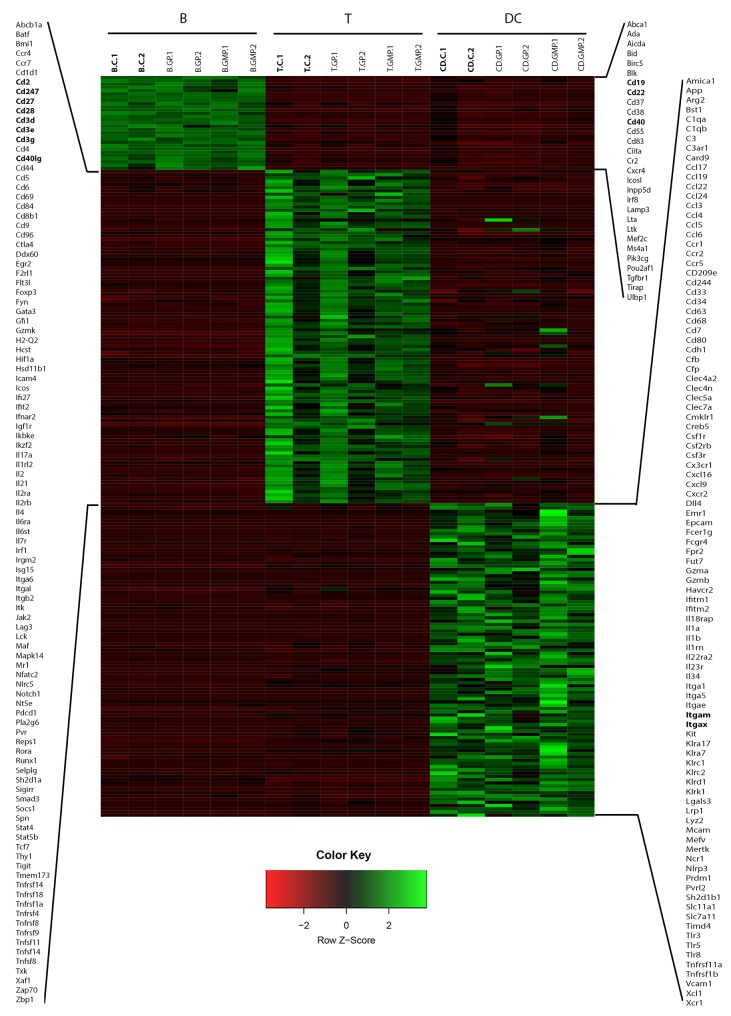
Heatmap compares gene expression profiles of upregulated genes in B-cells, T-cells and DCs. Z-scores in this heatmap show that a set of genes are upregulated specifically per cell type. Red indicates downregulation, green indicates upregulation, C denotes control, and GP and GMPs indicate treatment with particles. Data is presented in duplicate.

To determine whether we could find additional cell specific transcripts, we performed DEseq2 analysis between control B-cells, T-cells, and CD11c^+^ phagocytes. Our stringent cut-off criteria were the same as described earlier. The results reveal that there were a total of 396 differentially expressed genes. Among those transcripts, 29 genes were only upregulated in B-cells, 103 genes were only upregulated in T-cells, and 97 genes were only upregulated in CD11c^+^ phagocytes (**Fig. 5**). We also found that 63 differentially expressed genes were only downregulated in B-cells, 70 genes that were only downregulated in T-cells, and 34 genes that were only downregulated in DCs (**Fig. S4**). The data used in our representative heatmaps demonstrates the reproducibility of this method across all cell types under experimental conditions involving stimulation with GPs and GMPs.

The reported method here provides a reproducible approach to isolating high-quality RNA from PP, B-cells, T-cells and CD11c^+^ phagocytes which allowed the application of NanoString technology to determine changes in the transcriptional profiles of these cells. To achieve optimal results with this protocol, it is important to use an RNase inhibitor that is suited for PPs immediately after harvesting tissues. Careful cell sorting is also an important part of this method, as cells must be properly gated to ensure the highest cell purity during cell sorting. Lastly, the stringent data analysis that we describe will identify differentially expressed genes with high confidence. As an example of the utility of this method, we were able identify changes in genetic expression of GPs and GMPs β-glucan delivery vehicles that can target PP CD11c^+^ phagocytes. A major advantage of the presented method is that the investigator can perform intestinal immune profiling per cell type with any antigen or microbe of interest that is introduced into the intestinal mucosa.

### TROUBLESHOOTING

Possible problems and their troubleshooting solutions are listed in **Table 2**.

## Supplementary Material

Supplementary information **Figure S1.** Specific gene targets in all cell types are tested by RT-PCR.
**Figure S2.** Percent viability of PP single cell suspensions.
**Figure S3.** Stabilization of PP RNA is important for nanostring-based methods.
**Figure S4.** Heatmap compares gene expression profiles of downregulated genes in B-cells, T-cells and DCs.
Supplementary information of this article can be found online at
http://www.jbmethods.org/jbm/rt/suppFiles/246.

## Figures and Tables

**Table 1.** Differential gene expression of GPs and GMPs in DCs.

	GPs		GMPs	
	Fold change	Adjusted *P*	Fold change	Adjusted *P*
IL-22	NS*		21.8	1.31E-06
Dmbt1	NS*		10.9	0.000183
IL-1r1	2.5	0.16481	5.6	0.03035
Fn1	NS*		5.9	0.040187

*NS denotes no significant differential gene expression.

**Table 2.** Possible problems and their troubleshooting solutions.

Step #	Problems	Causes	Suggestions
2	Rapid RNA degradation	Adequate amounts of SUPERase·In RNase inhibitor were not added	Add suggested amounts of SUPERase·In RNase inhibitor
3	Rapid RNA degradation	PPs were not ground on ice	PPs must always be dissociated on ice
3.11	Cells clump before sorting	Small volumes of flow buffer	Start with a minimum of 1–2 ml of flow buffer as cells are filtered through
4	Rapid RNA degradation	Harsh cell lysis	Use a Spin-X column not a syringe
7.1	NanoString low input kit does not seem to be efficient	Very low levels of RNA	Estimate the minimum number of cycles needed for cDNA amplification, we used the RT-qPCR assay
